# Effects of neuroticism on suicide risk in major depressive disorder and bipolar disorder

**DOI:** 10.3389/fpsyt.2025.1527054

**Published:** 2025-05-19

**Authors:** Yujie Xing, Tian Li, Zhen Mao, Lei Zhao, Yue Sun, Qitong Jiang, Chuanyue Wang, Qijing Bo

**Affiliations:** ^1^ Beijing Key Laboratory of Mental Disorders, National Clinical Research Center for Mental Disorders & National Center for Mental Disorders, Beijing Anding Hospital, Capital Medical University, Beijing, China; ^2^ Advanced Innovation Center for Human Brain Protection, Capital Medical University, Beijing, China

**Keywords:** suicide risk, major depressive disorder, bipolar disorder, personality traits, neuroticism

## Abstract

**Background:**

Suicide is the primary cause of death in patients with major depressive disorder (MDD) or bipolar disorder (BD). Among various personality traits, neuroticism is particularly relevant to suicide risk. However, its role in MDD and BD has not been examined sufficiently. This study characterized neuroticism in patients with MDD or BD, and analyzed the association between neuroticism and suicide risk in these patients.

**Methods:**

This study collected demographic information and personality traits of MDD and BD patients. Group differences were assessed using t-tests, chi-square tests, and Mann-Whitney U tests. To identify factors associated with suicide risk, correlation analysis was first conducted, followed by bivariate and generalized ordered logistic regression for significant variables, including neuroticism. Sensitivity analyses were performed by progressively excluding potential confounders to evaluate the robustness of neuroticism’s effect. Additionally, simple mediation analyses using a bootstrap approach were conducted to examine whether depressive symptoms mediated the association between neuroticism and suicide risk in MDD and BD separately. A two-tailed *P* < 0.05 was considered statistically significant.

**Results:**

The study population comprised 88 MDD patients and 90 BD patients. Lifetime suicide risk was present in 39.3% of the study population. In the entire sample, neuroticism was significantly associated with both lifetime (*r* = 0.18, *P* = 0.018) and current suicide risk (*r* = 0.17, *P* = 0.024). In patients with MDD, through mediation analysis, neuroticism predicted both depressive symptom severity (B = 0.25, *P* < 0.001) and current suicide risk (B = 0.02, *P* = 0.022), while also indirectly influencing current suicide risk through depressive symptoms (B = 0.01, 95% CI = 0.01–0.02). In BD patients, neuroticism predicted depressive symptoms (B = 0.13, *P* = 0.002) but not current suicide risk (B < 0.01, *P* = 0.714), while depressive symptoms fully mediated this relationship (B = 0.06, *P* < 0.001).

**Conclusion:**

Neuroticism plays a significant role in influencing suicide risk among MDD and BD, through its effect on depressive symptoms. Interventions for neuroticism can reduce depressive symptoms and suicide risk. This highlights the necessity of identification and management of neuroticism in suicide prevention strategies.

## 1 Introduction

Affective disorders (ADs) are highly prevalent disorders all over the world. Over 163 million people had major depressive disorder (MDD) in 2017 (1). In 2019, an estimated 280 million individuals worldwide were affected by depression, while approximately 40 million were diagnosed with bipolar disorder (BD) (2). MDD and BD are serious ADs that negatively impact normal and social life. Given the high prevalence and psychiatric severity, the associated suicide risk poses a serious public health concern. At the same time, the pathogenesis of each disorder involves multiple factors and complex pathological mechanisms that remain unclear.

Suicide is a severe outcome of ADs, including ideation, attempt, and completion. Completed suicide is defined as an intentional self-directed injurious act with the goal of self-imposed fatality. A suicide attempt is a nonfatal act driven by the intent to die. Whether suicide is only attempted or completed, each is preceded by suicidal ideation, that is, thinking about or planning death. Suicidal ideation is a strong predictor of suicide attempts and completed suicides (3).

Both BD and MDD are associated with a high risk of suicide. For example, a study reported that approximately 30% of BD patients attempt suicide at least once in their lifetime (4). Another study found that approximately 20% of patients with first-episode MDD in youth attempted suicide (5). According to Miola et al. (6), type II BD carries a greater suicide risk than MDD. Furthermore, patients with recurrent depression and those in the early stage of a major depressive episode are at an increased risk of suicidal behavior (7). However, another study found that in 87 patients with ADs, suicide attempts were more severe in the later course of the illness. Notably, the risk of suicide among adolescent MDD patients has been increasing annually (8). The high rate of suicide among patients with ADs calls for greater awareness and intervention from clinicians.

Beyond ADs, additional predictors of suicide include genetic factors, personality traits, gender, quality of social network, personal and interpersonal difficulties, and stressful life events (9–12). Personality characteristics have considerable influence on the emergence of suicidal behavior (10, 13). Eysenck’s personality theory classifies personality traits as neuroticism, extraversion, and psychoticism. The Eysenck Personality Questionnaire (EPQ) is a self-reported questionnaire grounded in Eysenck’s personality theory. In the EPQ, the neuroticism score reflects levels of anxiety, depression, and self-doubt. The score for psychoticism indicates the degree of egocentricity, impulsivity, hostility, and antisocial behavior. The extraversion scale measures a continuum from introversion to extraversion, incorporating classical temperament types: phlegmatic (introverted-stable), melancholic (introverted-unstable), sanguine (extraverted-stable), and choleric (extraverted-unstable) (14).

Brezo et al. (15) reported that neuroticism scores may serve as indicators of suicidal risk. Neuroticism increases the risk of suicide in both general populations and individuals with ADs, and remains a significant predictor among women with ADs (16). Lower emotional stability, reflected by high neuroticism, is associated with rumination and depressive symptoms, both of which are strongly linked to increased suicide risk (17). This finding aligns with the results of Bi et al. (18), indicating that higher neuroticism increases the risk of suicide attempts among individuals with psychiatric disorders. Individuals with elevated neuroticism are more sensitive to negative emotions and stress, which may exacerbate depressive symptoms and contribute to increased risk of suicide. In MDD, neuroticism may worsen mental health by aggravating core depressive symptoms, including concentration difficulties and fatigue (19).Research indicates that patients with chronic MDD exhibit elevated risk of suicide and higher neuroticism scores (20).

Compared to MDD, the link between neuroticism and suicide risk in BD is more complex, as neuroticism remains a stable trait that persists even during remission (21). High neuroticism is associated with poor emotional regulation and heightened susceptibility to depressive episodes. In BD, it is also linked to cognitive impairments, including difficulties with decision-making, category fluency, and response inhibition. These deficits impair social functioning and contribute to elevated risk of suicide. In BD, high neuroticism is associated with suicidal ideation in the absence of prior suicidal behavior, and is linked to increased risk of new-onset suicidal tendencies (22). A previous study (23) reported that BD episodes with mixed features, particularly agitation, tend to result in suicidal behaviors. This susceptibility contributes to elevated rates of suicidal ideation and behaviors during major depression or mixed episodes (24). However, evidence linking neuroticism to suicide risk in BD is limited and less conclusive than in MDD. Negative urgency, a facet of impulsivity, has been linked to both suicidal ideation and neuroticism in BD, but its neural basis remains unclear (25). In a longitudinal study, Kamali et al. (26) found that baseline personality traits did not predict suicide attempts in BD. However, neuroticism emerged as a predictor of suicidal ideation over the course of illness.

In summary, neuroticism, which is linked to both depressive symptoms and suicide risk, contributes to the elevated suicide risk observed in individuals with MDD or BD. However, how neuroticism specifically influences suicide risk in MDD and BD remains unclear. This study aimed to explore the relationship between neuroticism and suicide risk by examining the mediating role of depressive symptoms. It further examined how neuroticism and depressive symptoms interact in both lifetime and current suicide risk, with the goal of generating clinically relevant insights for targeted intervention and prognosis.

## 2 Methods

### 2.1 Participants

This cross-sectional study was approved by the ethics committee of Beijing Anding Hospital, Capital Medical University. Informed consent was obtained from all participants or their guardians.

From September 2014 to January 2016, 214 patients were recruited from the in- and outpatient departments at Beijing Anding Hospital, Capital Medical University, Beijing, China. For inclusion in this study, each patient had to be between 16 to 60 years old, and have obtained at least 9 years of education. Potential subjects with any of the following were excluded: pre-existing or current organic brain disorder (e.g., dementia, epilepsy, and brain injury), intellectual disability, ADs caused by physical illness, other psychiatric disorders (e.g., schizophrenia and drug-induced disorders), and an inability to complete the assessments.

Finally, the total study population comprised 178 patients, of whom 88 were diagnosed with MDD, and 90 with BD. The MDD and BD diagnoses were established using the Structured Clinical Interview for DSM-IV Axis I-Patient Edition (SCID-I/P) (27).

### 2.2 Clinical assessments

In this study, all scales were scored by clinical psychiatrists with extensive experience who were specially trained to administer standardized instruments, including the SCID-I/P, HAMD-17, and EPQ. Before formal assessments commenced, evaluators independently rated a set of training cases, with any discrepancies resolved through consensus discussions. To maintain reliability throughout the study, regular quality control measures were implemented. Specifically, 10% of the assessments were randomly selected for re-evaluation by an independent rater who was blinded to the original scores. Additionally, periodic refresher training sessions were conducted to mitigate rater drift.

### 2.3 Lifetime suicide risk

This study assessed 3 dimensions of lifetime suicide risk: ideation, attempt, and behavior. These aspects were assessed qualitatively based on the self-reported responses of the subjects (with MDD or BD) to the following questions in the SCID-I/P: “Have you ever had or do you currently have suicidal ideation?”, “Have you ever attempted suicide in the past or recently?”, and “Have you ever committed or are you currently committing suicidal behavior?”. Utilizing structured clinical interviews with the SCID-I/P allows for the documentation of all suicide-related experiences throughout a patient’s lifetime, facilitated an in-depth exploration of the context and motivations for each suicidal behavior. This comprehensive lifetime assessment significantly enhanced the reliability and consistency of the data. In this study, suicidal behavior was defined as self-injurious acts performed with a clear intent to die. Acts of non-suicidal self-injury were excluded from this classification.

### 2.4 Current suicide risk

Current suicide risk was assessed using the item concerning suicide in the 17-item Hamilton Depression Rating Scale (HAMD-17). HAMD-17 is widely used to assess the severity of depression and the major symptoms of MDD, including mood, feelings of guilt, suicidal ideation, insomnia, anxiety, weight loss, and somatic symptoms (28). Researchers consider this scale to be accurate, reliable, and valid when applied in clinical studies (29). The present study stratified an individual’s current suicide risk (i.e., the suicide risk at present and in the past 1 week) into the following 5 levels, from mild to severe: “Absent”; “Feels life is not worth living”; “Wishes he/she were dead or any thoughts of possible self-inflicted death”; “Ideas or gestures of suicide”; and “Attempts at suicide” (28, 30, 31).

### 2.5 Eysenck personality questionnaire

The EPQ is a self-reported personality test covering mainly three personality domains, each of which is measured on a continuum: psychoticism, extraversion, and neuroticism (EPQ-P, EPQ-E, and EPQ-N, respectively). Altogether, the EPQ comprises 88 items, with 23, 24, and 21 items applied to the psychoticism, extraversion, and neuroticism domains, respectively. An additional lie scale comprises 20 items, in which lying is determined when the respondent identifies rarely performed desirable acts as typical, and denies common non-desirable acts (32). The respondent answers “yes” or “no” to each question. The EPQ is considered highly reliable and valid, with no differences in gender, and is widely used in medical research (33, 34).

### 2.6 Statistical analysis

IBM SPSS Statistics version 23.0 for Windows (SPSS, Chicago, IL, USA) was employed for data analyses. For the evaluation of demographic and other patient characteristics, the *t*-test was used to examine the following continuous variables in 2 independent samples: age; years of education; and the EPQ-P, EPQ-E, EPQ-N, EPQ-L and PHQ-9 scores. The chi-square test was used to compare the dichotomous variables gender, diagnosis, residential and marital status and employment. The Mann-Whitney *U* test was applied to test for disease duration in 2 independent samples in which the distribution was not normal. Spearman correlation analysis was used to examine the associations between variables and lifetime or current suicide risk. Significant variables will be included in bivariate or generalized ordered logistic regression analyses. Sensitivity analyses were conducted to confirm the robustness of variables in predicting suicide risk outcomes. A stepwise exclusion method was used to progressively remove confounding variables and observe changes in the effect of EPQ-N scores across models. Simple mediation models were employed to assess the mediating role of PHQ-9 between neuroticism and current suicide risk. The level of significance was set at *P* < 0.05 (2-tailed).

## 3 Results

### 3.1 Demographic and clinical characteristics

Of the initial 214 recruits with MDD or BD, 36 were excluded due to failure to complete the assessments. Thus, the study population comprised 178 patients with fully completed assessments (**Table 1**). There were 99 (55.6%) men and 79 (44.4%) women. Among them, 108 (60.7%) and 70 (39.3%) participants were judged to have no or some lifetime suicide risk, respectively. The 70 patients with a lifetime risk of suicide had a significantly longer disease duration than did the 108 patients without a lifetime risk of suicide (Z = –3.82, *P* < 0.001). Patients living alone had a significantly greater lifetime risk of suicide compared to those living with co-residents (*χ²* = 7.94, *P* = 0.005). The groups with and without lifetime suicide risk were statistically comparable in terms of age, years of education, gender ratio, marital status, employment, and PHQ-9 scores.

**Table 1:** Demographics and characteristics of patients with ADs without and with lifetime suicide risk.

Characteristics	Overall	Lifetime suicide risk		Test Statistic	P Value
		Absent	Present		
Subjects, *n*	178	108	70		
				*t/Z/χ^2^*	*P*
Age, y	31.80 ± 10.81	31.02 ± 10.81	33.01 ± 10.77	–1.21	0.230
Education, y	13.35 ± 3.12	13.10 ± 3.02	13.73 ± 3.25	–1.31	0.191
EPQ-P	51.24 ± 9.78	50.13 ± 8.90	52.06 ± 10.84	–1.90	0.059
EPQ-E	47.53 ± 11.55	48.20 ± 12.04	46.49 ± 10.75	0.10	0.338
EPQ-N	54.37 ± 13.50	52.51 ± 13.68	57.24 ± 12.79	–2.31	0.022*
EPQ-L	44.83 ± 10.27	45.07 ± 9.71	44.47 ± 11.13	0.38	0.704
PHQ-9	9.50 ± 6.81	8.83 ± 6.44	10.56 ± 7.26	-1.62	0.108
Duration, median (IQR), months	57.50 (20.75, 96.00)	45.00 (18.00, 97.00)	60.50 (24.00, 96.00)	–3.82	<0.001**
Male sex, *n* (%)	99 (55.61)	64 (59.26)	35 (50.00)	1.48	0.225
Residential status, living wo, *n* (%)	163 (91.57)	104 (96.30)	59 (84.29)	7.94	0.005*
MDD, *n* (%)	88 (49.44)	57 (52.78)	31 (44.29)	1.23	0.268
Occupation, Employed, n (%)	98 (55.06)	57 (47.22)	29 (41.43)	0.58	0.448
Marriage state, married, n (%)	79 (44.38)	45 (41.67)	34 (48.57)	2.13	0.588

*P < 0.05; **P < 0.01.

ADs, affective disorders; EPQ-P, Eysenck Personality Questionnaire - Psychoticism; EPQ-E, Eysenck Personality Questionnaire - Extraversion; EPQ-N, Eysenck Personality Questionnaire - Neuroticism; EPQ-L, Eysenck Personality Questionnaire – Lie; PHQ-9, Patient Health Questionnaire-9; IQR, interquartile range; living wo, living with others; MDD, major depressive disorder.

The EPQ-N scores of the patients with a lifetime risk of suicide were significantly higher than those of patients without a lifetime suicide risk (*t* = –2.31, *P* = 0.022; **Table 1**). However, the EPQ-P and EPQ-E scores did not significantly differ between the two groups of patients (*t* = –1.90, *P* = 0.059 and *t* = 0.10, *P* = 0.338, respectively). There was no statistical significance in EPQ-L scores between the two groups (*t* = 0.38, *P* = 0.704), indicating that the results of the main scales (EPQ-E, P, and N) are likely more reliable and less influenced by socially desirable or masked responses.

### 3.2 Lifetime suicide risk and personality traits

In the overall population, the lifetime suicide risk was positively associated with the EPQ-N score (*r* = 0.18, *P* = 0.018; **Table 2**) and disease duration (*r* = 0.29, *P* < 0.001). A significant correlation was found between residential status and lifetime suicide risk (*r* = 0.21, *P* = 0.005), with those living with others having a lower risk. Specifically, in the MDD group, the lifetime suicide risk was positively associated with disease duration (*r* = 0.37, *P* < 0.001). Lifetime suicide risk was positively correlated with residential status (*r* = 0.28, *P* = 0.008) in the BD group.

**Table 2:** Associations between potential influencing factors and lifetime suicide risk in ADs.

Potential influencing factors	Overall		MDD		BD	
	*r*	*P*	*r*	*P*	*r*	*P*
Age	0.10	0.169	0.12	0251	0.13	0.211
Years of education	0.10	0.195	0.14	0.203	0.06	0.550
EPQ-P	0.14	0.064	0.16	0.144	0.12	0.279
EPQ-E	–0.06	0.394	–0.01	0.277	–0.03	0.750
EPQ-N	0.18*	0.018	0.19	0.085	0.18	0.088
Disease duration	0.29**	<0.001	0.37**	<0.001	0.17	0.104
PHQ-9	0.11	0.138	0.20	0.062	0.10	0.427
Residential status	0.21**	0.005	0.19	0.083	0.28**	0.008
Occupation	-0.04	0.558	-0.03	0.789	-0.05	0.633
Marriage	-0.07	0.383	-0.01	0.973	-0.14	0.194
Gender	0.09	0.227	0.06	0.613	0.15	0.151

*P < 0.05; **P < 0.01.

ADs, affective disorders; MDD, major depressive disorder, BD, bipolar disorder; EPQ-P, Eysenck Personality Questionnaire - Psychoticism; EPQ-E, Eysenck Personality Questionnaire - Extraversion; EPQ-N, Eysenck Personality Questionnaire - Neuroticism; PHQ-9, Patient Health Questionnaire -9.

Binary logistic regression analysis showed that for the overall population, the following were independent predictors of lifetime suicide risk (**Table 3**): living alone (odds ratio [OR] = 4.59, 95% confidence interval [CI] = 1.34–15.73, *P* = 0.015) and longer disease duration (OR = 1.01, 95% CI = 1.00–1.01, *P* = 0.001), after adjusting for all variables. However, the impact of high neuroticism on lifetime suicide risk was not statistically significant (*P* = 0.187). In sensitivity analyses, after stepwise exclusion of other variables, the EPQ-N score was found to be a significant predictor of lifetime suicide risk (OR = 1.03, 95% CI = 1.00–1.05, *P* = 0.024). This suggests that while the EPQ-N was a significant predictor of lifetime suicide risk when included as the sole variable, its effect became non-significant when adjusted for variables such as disease duration and residential status. For patients with MDD, both longer disease duration (OR = 1.02, 95% CI = 1.01–1.03, *P* < 0.001) and higher PHQ-9 scores (OR = 1.08, 95% CI = 1.00–1.16, *P* = 0.044) was independent predictor of lifetime suicide risk. Although residential status correlated with lifetime suicide risk in the BD group, the association did not reach statistical significance.

**Table 3:** Results of binary logistic regression analysis of lifetime suicide risk.

Dependent	Independent	B	SE	Wald	Exp(B)	*P*	95% CI for Exp(B)	
							Lower	Upper
ADs wo/w lifetime suicide risk	Residential status	1.52	0.63	5.88	4.59	0.015	1.34	15.73
	Disease duration	<0.01	<0.01	10.95	1.01	0.001	1.00	1.01
	EPQ-N	0.03	0.01	5.10	1.03	0.024	1.00	1.05
MDD wo/w lifetime suicide risk	Disease duration	0.02	<0.01	12.91	1.02	<0.001	1.01	1.03
	PHQ-9	0.07	0.04	4.06	1.08	0.044	1.00	1.16

ADs, affective disorders; wo/w, without/with; SE, standard error; CI, confidence interval; EPQ-N, Eysenck Personality Questionnaire - Neuroticism; MDD, major depressive disorder; PHQ-9, Patient Health Questionnaire-9.

### 3.3 Current suicide risk and personality traits

Spearman correlation analysis showed that in the overall population, the current suicide risk was inversely associated with the EPQ-E score [*r* = –0.19, *P* = 0.010; **Table 4**)], but positively associated with EPQ-N score and residential status (*r* = 0.17, *P* = 0.024 and *r* =0.19, *P* =0.010, respectively. The positive correlation between PHQ-9 scores and current suicide risk was notably significant, with a moderate level of correlation (*r* = 0.46, *P* < 0.001). Further subgroup analyses revealed that among patients with MDD, the current suicide risk was inversely associated with age (*r* = –0.23, *P* = 0.033) and positively associated with EPQ-N and PHQ-9 score (*r* = 0.24, *P* = 0.026 and *r* =0.45, *P* < 0.001, respectively). In BD group, current suicide risk was positively correlated with residential status (*r* = 0.41, *P* < 0.001) and PHQ-9 score (*r* = 0.49, *P* < 0.001), negatively correlated with the EPQ-E score (*r* = –0.21, *P* = 0.043), but showed no significant association with the EPQ-N score (*r* = 0.10, *P* = 0.355).

**Table 4:** Associations between potential influencing factors and current suicide risk.

Potential influencing factors	Overall		MDD		BD	
	*r*	*P*	*r*	*P*	*r*	*P*
Age	–0.11	0.156	–0.23*	0.033	–0.02	0.831
Years of education	–0.04	0.564	–0.04	0.717	–0.05	0.654
EPQ-P	0.09	0.231	0.05	0.678	0.15	0.164
EPQ-E	–0.19*	0.010	–0.17	0.117	–0.21*	0.043
EPQ-N	0.17*	0.024	0.24*	0.026	0.10	0.355
PHQ-9	0.46**	<0.001	0.45**	<0.001	0.49**	<0.001
Residential status	0.19*	0.010	0.03	0.805	0.41**	<0.001
Occupation	0.13	0.087	0.13	0.242	0.13	0.222
Marriage	0.08	0.277	0.10	0.347	0.08	0.466
Disease duration	0.06	0.468	<0.01	0.983	0.09	0.393
Gender	<0.01	0.972	–0.11	0.323	0.11	0.314

*P < 0.05; **P < 0.01.

MDD, major depressive disorder; BD, bipolar disorder; EPQ-P, Eysenck Personality Questionnaire - Psychoticism; EPQ-E, Eysenck Personality Questionnaire - Extraversion; EPQ-N, Eysenck Personality Questionnaire - Neuroticism; PHQ-9, Patient Health Questionnaire-9.

The results of generalized ordered logistic regression analyses showed that PHQ-9 scores significantly influenced current suicide risk in both the overall ADs (OR = 1.19, 95% CI = 1.10–1.28, *P* < 0.001; **Supplementary Table S1**) and subgroups of MDD (OR = 1.21, 95% CI = 1.10–1.33, *P* < 0.001) and BD (OR = 1.25, 95% CI = 1.09–1.43, *P* = 0.001). Among BD patients, residential status and disease duration significantly influenced current suicide risk. Having co-residents was a protective factor against current suicide risk in BD patients (OR < 0.01, 95% CI = 0.01–0.90, *P* = 0.001), while longer disease duration was linked to heightened vulnerability, it did not have a significant impact on current suicide risk (OR = 1.01, 95% CI = 0.99–1.03, *P* = 0.039).

### 3.4 The mediating role of depressive symptoms

**Figure 1:**
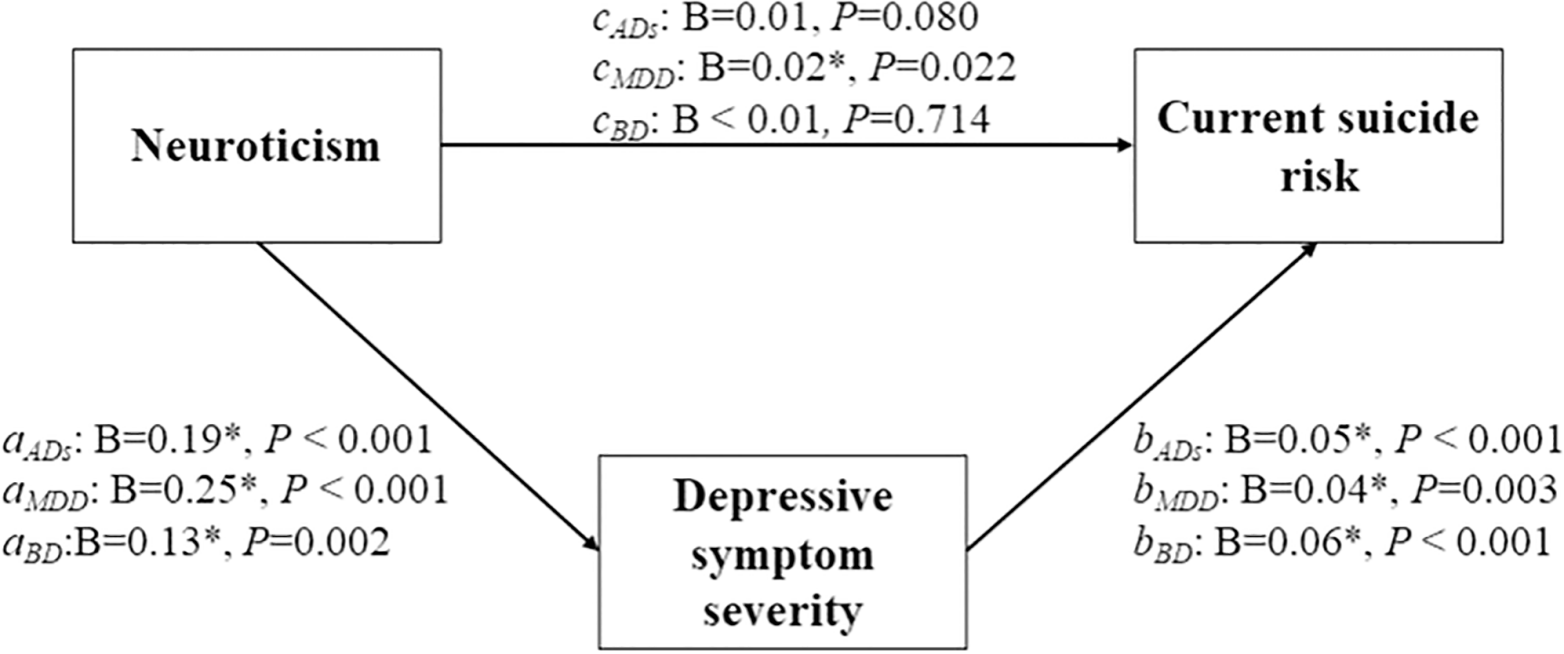
Mediation effect of depression severity in Neuroticism influencing current suicide risk. In MDD, neuroticism influences current suicide risk both directly and indirectly through depressive symptoms (*a_MDD_*: B=0.25*, *P* < 0.001; *b_MDD_*: B=0.04*, *P*=0.003; *c_MDD_*: B=0.02*, *P*=0.002). In BD, neuroticism influences current suicide risk mainly through depressive symptoms, not directly (*a_BD_*: B=0.13*, *P*=0.002; *b_BD_*: B=0.06*, *P* < 0.001; *c_BD_*: B < 0.01, *P*=0.714). For ADs as a whole, the overall effect is predominantly indirect via depressive symptoms (*a*_ADs_: B=0.19*, *P* < 0.001; *b*_ADs_: B=0.05*, *P* < 0.001; *c*_ADs_: B=0.01, *P*=0.080).

Mediation analysis demonstrated that depressive symptoms acted as a key mediator in the relationship between neuroticism and current suicide risk (**Figure 1**). In the overall ADs, neuroticism significantly predicted depressive symptom severity (B = 0.19, *P* < 0.001; **Supplementary Table S2**), which in turn predicted current suicide risk (B = 0.05, *P* < 0.001). Although the total effect of neuroticism on current suicide risk approached significance (B = 0.01, *P* = 0.080), its direct effect was not statistically significant (B < 0.01, *P* = 0.680). Bootstrap tests confirmed that depressive symptoms fully mediated the relationship between neuroticism and current suicide risk (B = 0.01, 95% CI = 0.01–0.02). In MDD, neuroticism was a strong predictor of depressive symptoms (B = 0.25, *P* < 0.001; **Table 5**), which subsequently predicted current suicide risk (B = 0.04, *P* = 0.003). A significant total effect of neuroticism on current suicide risk was observed (B = 0.02, *P* = 0.022), its direct influence failed to reach statistical significance (B < 0.01, *P* = 0.500). Bootstrap tests showed that depressive symptoms partially mediated this relationship, with an indirect effect of (B = 0.01, 95% CI = 0.01–0.02). Similarly, in the BD group, neuroticism predicted depressive symptoms (B = 0.13, *P* = 0.002; **Supplementary Table S3**), which also strongly predicted current suicide risk (B = 0.06, *P* < 0.001). However, neither the total effect (B < 0.01, *P* = 0.714) nor the direct effect (B = -0.01, *P* = 0.335) of neuroticism on current suicide risk was significant. The full mediation of depressive symptoms in the neuroticism-suicide risk relationship was confirmed by bootstrap analyses, showing an indirect effect of (B = 0.01, 95% CI = 0.01–0.02).

**Table 5:** Results of the mediating effect of depression severity in neuroticism influencing current suicide risk in MDD.

Effect	B	SE(B)/BootSE	*t*	*P*	LLCI/BootLLCI	ULCI/BootULCI
a: Neuroticism -> depression severity	0.25	0.06	4.34	<0.001	0.14	0.36
b: depression severity -> current suicide risk	0.04	0.01	3.75	0.003	0.02	0.07
c (total): Neuroticism -> current suicide risk	0.02	0.01	2.33	0.022	<0.01	0.03
c’ (direct): Neuroticism -> current suicide risk	<0.01	0.01	0.67	0.500	-0.01	0.02
ab (indirect): Neuroticism -> depression severity -> current suicide risk	0.01	<0.01	N/A	N/A	0.01	0.02

MDD, major depressive disorder; SE, standard error; LLCI, Lower Level Confidence Interval; BootLLCI, Bootstrap Lower Level Confidence Interval; ULCI, Upper Level Confidence Interval; BootULCI, Bootstrap Upper Level Confidence Interval; N/A, Not Applicable.

## 4 Discussion

This study examined the role of personality traits, particularly neuroticism, in the risk of suicide among patients with ADs, based on qualitative information concerning suicide risk, the suicide item of HAMD-17, and the EPQ. Across multiple analyses, neuroticism demonstrated a consistent association with suicide risk. Neuroticism showed a significant association with both lifetime and current suicide risk in patients with ADs. In univariate analyses, lifetime suicide risk was significantly associated with neuroticism; however, this effect was no longer significant after controlling for covariates. Mediation analyses showed that the effect of neuroticism on current suicide risk was mediated by depressive symptoms. In MDD patients, neuroticism contributed to an increased current suicide risk. The findings underscore neuroticism as a significant driver of suicide risk, both indirectly through depressive symptoms and directly. This effect is most pronounced in the context of heightened depressive symptoms.

Personality traits are unique and inherent to everyone and influence emotions, judgment, decisions, and behavior. According to Eysenck’s theory of personality, neuroticism is a stable personality trait (14). Thus, a high EPQ-N score suggests a persistent tendency experience a range of negative emotional states, including anxiety, frustration, envy, hopelessness, and loneliness, compared to individuals with a low score. The present study extends prior findings on the association between neuroticism and suicide risk, by clarifying its differential influences in MDD and BD.

Neuroticism, as measured by the EPQ-N, was elevated in participants with ADs who reported both lifetime and current risk of suicide. Patients with ADs and high EPQ-N scores were more likely to report lifetime suicide risk. A retrospective cohort study reported that high levels of neuroticism in adolescents with ADs may increase the risk of suicide in adulthood by promoting maladaptive behaviors (35). Su et al. suggested that a high level of neuroticism was an independent risk factor for both suicidal ideation and attempt. In addition, the combination of elevated neuroticism and reduced extraversion may characterize a subgroup of patients at particularly high risk for suicide (36). Another study identified neuroticism as a potential marker of chronic suicide risk (37). This study builds on previous findings by further clarifying the contribution of neuroticism to lifetime suicide risk in patients with ADs. Neuroticism predicted suicide risk in univariate analyses, but this effect was attenuated after controlling for factors such as living alone and prolonged illness. This supports the stress-vulnerability hypothesis (38), suggesting that neuroticism, as a biological vulnerability, plays a stronger role under high-stress conditions and a diminished role when stressors are minimal.

This study further showed that, among patients with MDD, high EPQ-N scores were significantly associated with current suicide risk. This result is consistent with previous studies (10, 39). Acute episodes of MDD are typically characterized by persistent low mood, lack of pleasure and interest, sleep and appetite disturbances, low energy, feelings of worthlessness, and, in severe cases, cognitive dysfunction, including memory deficits (40–42). Severe or persistent MDD may impair patients’ social functioning (43–45), leading to increased social isolation and heightened suicide risk. This cross-sectional study identified neuroticism as a contributor to current suicide risk, partly through depressive symptoms. The severity of depressive symptoms was evaluated in accordance with each participant’s clinical status at the time of assessment, encompassing both acute depressive episodes and remission. These observations indicate that the impact of neuroticism on current suicide risk is not restricted to periods of active depressive symptomatology. Rather, its influence appears to extend across various stages of illness. These findings support the view that neuroticism is a stable personality trait whose influence on current suicide risk endures throughout the course of MDD. Furthermore, neuroticism contributes directly to suicide risk and exacerbates depressive symptoms, which in turn further increase suicide risk in patients with MDD. According to the mediator-moderator model (46), neuroticism influences suicide risk by affecting depressive symptoms. As a mediator, depressive symptoms help explain the relationship between external stressors and internal personality traits. For example, individuals with high neuroticism tend to experience worsening depressive symptoms under stress or negative events, often accompanied by persistent difficulties in emotion regulation and ineffective coping strategies. This pattern of cognitive distortion, including over-interpretation and catastrophizing, further amplifies suicide risk in these patients.

Elevated neuroticism has also been identified as a predictor of BD symptoms, in both one-dimensional models (bipolarity) and two-dimensional models (mania and depression) (47). Although the suicide rate is high in BD, most cases occur during depressive or mixed episodes (48), underscoring the contribution of depressive symptoms to suicide risk in this population. One key finding of this study is that neuroticism indirectly increases suicide risk by exacerbating depressive symptoms, offering new insight into the suicide risk model in BD. The impact of neuroticism in BD may change over time. In particular, manic and depressive episodes could further shape its influence. Future research should further investigate how neuroticism interacts with clinical features, such as depressive episodes.

This study supports earlier findings regarding the relationship between selected psychological factors and suicide risk. In patients with MDD, disease duration was significantly longer in those with a reported lifetime risk of suicide compared to those without. Studies have revealed that even during remission, patients with chronic depression remain vulnerable to relapse and suicide, particularly as illness duration increases (49). Similarly, Pan et al. (50) found that the high risk of suicidal thoughts and behaviors was associated with the duration of the illness. One possible explanation is that the longer course of illness contributes a decline in social functioning and self-worth, which may, in turn, increase suicidal ideation. This study found that age was significantly associated with current suicide risk. Melhem et al. described that younger patient with depressive symptoms had a higher risk of suicide (51). However, Grav et al. (52) stated that older patients were more likely to exhibit suicidal behavior—a pattern not observed in the current study. In older adults, suicide risk may be related to bereavement, and living alone (53), whereas in younger individuals, it is more often linked to family conflict and impulsivity (54). Living alone often lacks social support, which significantly contributes to increased suicide risk. Feelings of loneliness, isolation, and hopelessness, intensified by living alone, are key drivers of suicidal ideation and behavior. The lack of social support reinforces these emotions and increases suicide risk (55).

The findings of this study suggest that high neuroticism is associated with heightened suicide risk. Neuroticism contributes to current suicide risk through different mediation pathways in MDD and BD, primarily via depressive symptoms. In MDD, neuroticism is associated with current suicide risk through both direct and indirect pathways. Pharmacological treatment remains the first-line approach for managing depressive symptoms. Nevertheless, additional benefit may be gained by combining medical treatment with personality interventions for individuals with high levels of neuroticism. Resilience training, targeted cognitive-behavioral therapy, and mindfulness may be effective strategies for addressing neuroticism. Integrating standard medical and personality-based interventions may lead to improve emotional regulation and reduce suicide vulnerability. In contrast, managing depressive symptoms should be prioritized in BD patients with high neuroticism to effectively address current suicide risk. This can be achieved through mood stabilization, and the reduction of acute symptoms. Meanwhile, by improving emotional regulation and preventing depressive recurrence, personality-informed interventions may offer protection against current suicide risk. Moving forward, these distinctions may inform the development of targeted suicide prevention strategies adapted to individual risk profiles.

A deeper knowledge of its inherent pathological systems may be obtained from multi-omics approaches. This could help identify individual vulnerability traits. It may also support the development of a robust framework for precise risk prediction and targeted interventions.

This study is subject to several limitations. The cross-sectional design of the study prevented the collection of follow-up data on individual trajectories. In addition, the potential effect of antipsychotics medication on suicide risk could not be evaluated. Such evaluations would require longitudinal studies to better clarify the relationship between neuroticism and suicide risk. The focus of this study was on the association between neuroticism and suicide risk—both lifetime and current, without distinguishing between suicide attempts and suicidal ideation. Analyzing the association between neuroticism and either suicide attempts or suicidal ideation was beyond the scope of this study. Future studies should incorporate multiple follow-up assessments to capture changes in depressive and manic symptoms over time. Additionally, a broader age range was included to improve sample diversity. Despite phenotypic differences in ADs across age groups, including participants aged 16 years and older offers substantial value for research. Future studies should expand the sample size, with particular attention to adolescents and young adults. A final limitation is that all participants were from a Chinese population, potentially restricting the generalizability of the findings. Cultural differences in emotional expression, help-seeking, and attitudes toward suicide could influence the observed associations. Future studies should examine the consistency of these patterns across culturally diverse populations.

## 5 Conclusion

This study explored the relationship between neuroticism and suicide risk in MDD and BD. The findings demonstrated a significant association between neuroticism and both lifetime and current suicide risk in patients with ADs. Mediation analyses further indicated the neuroticism contributes current suicide risk, by intensifying depressive symptoms, both directly and indirectly. Clinicians are encouraged to evaluate neuroticism to develop personalized interventions and enhance suicide prevention measures.

## Data Availability

The raw data supporting the conclusions of this article will be made available by the authors, without undue reservation.
