# Supplementary material for: Effects of neuroticism on suicide risk in major depressive disorder and bipolar disorder
Source: Front Psychiatry. 2025 May 19;16:1527054. doi: 10.3389/fpsyt.2025.1527054 (PMC12127354; doi:10.3389/fpsyt.2025.1527054)
Supplement: Supplementary file 1 [file Table1.docx]

Supplementary Material

# Supplementary Tables

**Supplementary Table S1.** Results of generalized ordered logistic regression analysis of current suicide risk

|  |  |  | |  |  | **95% CI for Exp(B)** | |
| --- | --- | --- | --- | --- | --- | --- | --- |
| **Dependents** | **Independents** | **B** | **SE** | **EXP(B)** | ***P*** | **Lower** | **Upper** |
| ADs wo/w current suicide risk | PHQ-9 | 0.17 | 0.04 | 1.19 | <0.001 | 1.10 | 1.28 |
| MDD wo/w current suicide risk | PHQ-9 | 0.19 | 0.05 | 1.21 | <0.001 | 1.10 | 1.33 |
| BD wo/w current suicide risk | Residential status | -5.62 | 1.62 | ＜0.01 | 0.001 | ＜0.01 | 0.90 |
|  | Disease duration | 0.01 | <0.01 | 1.01 | 0.039 | 0.99 | 1.03 |
|  | PHQ-9 | 0.22 | 0.07 | 1.25 | 0.001 | 1.09 | 1.43 |

Abbreviations: ADs, affective disorders; wo/w, without/with; PHQ-9, Patient Health Questionnaire-9; SE, standard error; CI, confidence interval; MDD, major depressive disorder; BD, bipolar disorder

**Supplementary Table S2.** Results of the mediating effect of depression severity in Neuroticism influencing current suicide risk in ADs

| **Effect** | **B** | **SE(B)/** **BootSE** | ***t*** | ***P*** | **LLCI/** **BootLLCI** | **ULCI/** **BootULCI** |
| --- | --- | --- | --- | --- | --- | --- |
| a: Neuroticism -> depression severity | 0.19 | 0.04 | 5.51 | <0.001 | 0.12 | 0.26 |
| b: depression severity -> current suicide risk | 0.05 | 0.01 | 5.62 | <0.001 | 0.03 | 0.06 |
| c (total): Neuroticism -> current suicide risk | 0.01 | <0.01 | 1.74 | 0.080 | <0.01 | 0.02 |
| c’ (direct): Neuroticism -> current suicide risk | <0.01 | <0.01 | -0.41 | 0.680 | -0.01 | 0.01 |
| ab (indirect): Neuroticism -> depression severity -> current suicide risk | 0.01 | <0.01 | N/A | N/A | 0.01 | 0.02 |

Abbreviations: ADs, affective disorders; SE, standard error; LLCI, Lower Level Confidence Interval; BootLLCI, Bootstrap Lower Level Confidence Interval; ULCI, Upper Level Confidence Interval; BootULCI, Bootstrap Upper Level Confidence Interval; N/A, Not Applicable

**Supplementary Table S3.** Results of the mediating effect of depression severity in Neuroticism influencing current suicide risk in BD

| **Effect** | **B** | **SE(B)/** **BootSE** | ***t*** | ***P*** | **LLCI/** **BootLLCI** | **ULCI/** **BootULCI** |
| --- | --- | --- | --- | --- | --- | --- |
| a: Neuroticism -> depression severity | 0.13 | 0.04 | 3.17 | 0.002 | 0.05 | 0.21 |
| b: depression severity -> current suicide risk | 0.06 | 0.01 | 4.21 | <0.001 | 0.03 | 0.09 |
| c (total): Neuroticism -> current suicide risk | <0.01 | 0.01 | 0.37 | 0.714 | -0.01 | 0.01 |
| c’ (direct): Neuroticism -> current suicide risk | -0.01 | 0.01 | -0.97 | 0.335 | -0.02 | 0.01 |
| ab (indirect): Neuroticism -> depression severity -> current suicide risk | 0.01 | <0.01 | N/A | N/A | 0.01 | 0.02 |

Abbreviations: BD, bipolar disorder; SE, standard error; LLCI, Lower Level Confidence Interval; BootLLCI, Bootstrap Lower Level Confidence Interval; ULCI, Upper Level Confidence Interval; BootULCI, Bootstrap Upper Level Confidence Interval; N/A, Not Applicable
